# MCM3AP Is Transcribed from a Promoter within an Intron of the Overlapping Gene for GANP

**DOI:** 10.1016/j.jmb.2010.12.035

**Published:** 2011-02-25

**Authors:** Vihandha O. Wickramasinghe, Paul I.A. McMurtrie, Jackie Marr, Yoko Amagase, Sarah Main, Anthony D. Mills, Ronald A. Laskey, Yoshinori Takei

**Affiliations:** 1 MRC Cancer Cell Unit, Hutchison/MRC Research Centre, Hills Road, Cambridge CB2 0XZ, UK; 2 Institute for Innovative NanoBio Drug Discovery and Development, Graduate School of Pharmaceutical Sciences, Kyoto University, 46-29 Yoshida-Shimo-Adachi-cho, Sakyo-ku, Kyoto 606-8501, Japan

**Keywords:** MCM3AP, MCM3 acetylating protein, TNFα, tumour necrosis factor α, 5′RACE, 5′ rapid amplification of cDNA ends, IL6, interleukin-6, gene structure, organisation

## Abstract

MCM3 acetylase (MCM3AP) and germinal-centre associated nuclear protein (GANP) are transcribed from the same locus and are therefore confused in databases because the MCM3 acetylase DNA sequence is contained entirely within the much larger GANP sequence and the entire MCM3AP sequence is identical to the carboxy terminus of GANP. Thus, the MCM3AP and GANP genes are read in the same reading frame and MCM3AP is an N-terminally truncated region of GANP. However, we show here that MCM3AP and GANP are different proteins, occupying different locations in the cell and transcribed from different promoters. Intriguingly, a promoter for MCM3AP lies within an intron of GANP. This report is an interesting example in nature of two separate gene products from the same locus that perform two entirely different functions in the cell. Therefore, to avoid further confusion, they should now be referred to as separate but overlapping genes.

### Introduction

MCM3 acetylating protein (MCM3AP) was previously identified in a yeast two-hybrid screen for human proteins that interacted with human DNA replication protein MCM3.^1^ MCM3AP can acetylate MCM3 weakly *in vivo* and shows homology to the Gcn5-related *N*-acetyltransferase superfamily including acetyl CoA binding sites.^2^ Overexpressed MCM3AP is an inhibitor of DNA replication initiation.^2,3^ It can shuttle between the nucleus and cytoplasm and accumulate in the nucleus in an MCM3-dependent manner.^3^ Following the discovery of MCM3AP, it was subsequently shown that the nucleotide sequence for MCM3AP is completely contained within the 3′ region of the sequence of germinal-centre associated nuclear protein (GANP),^4^ a  210-kDa protein that is upregulated in B cells and also in a variety of lymphomas.^5^ Therefore, it was suggested that MCM3AP may be a splice variant of GANP,^4^ and this view prevails in databases.

GANP has been proposed to have a role in the immune response. Thus, mice made deficient for GANP in immune cells showed reduced affinity maturation of antibodies against T-cell-dependent antigens and a lower frequency of variable region somatic mutation.^6^ However, we have recently shown that GANP has a more general role; namely, it is required for efficient mRNA export from the nucleus of mammalian cells.^7^ GANP depletion results in the nuclear accumulation of poly(A)+RNA, and it interacts directly with the major mRNA export factor NXF1 through its N-terminal FG repeat domain, which has extensive homology to nuclear pore proteins. Therefore, we have proposed that GANP may have a role in targeting mRNPs containing NXF1 to nuclear pores.^7^ In addition, GANP contains a domain that is homologous to Sac3, a component of the yeast mRNA export machinery.^8^ This Sac3 homology domain is present in many mammalian proteins, and it has been suggested to mediate protein–protein interactions within multi-protein complexes.^9,10^ Recently, it has been shown that GANP contains a conserved CID motif or Cdc31 interaction motif. In yeast, the Sac3 CID motif contributes to the targeting of Sac3 to the nuclear periphery and provides a scaffold within a transcription–export complex, TREX-2, to facilitate the coupling of transcription and mRNA export.^11^

In this study, we show that MCM3AP can be transcribed independently of GANP, consistent with GANP and MCM3AP functioning separately. Databases and literature on the genes encoding two overlapping proteins are confused and partly incorrect. MCM3AP is encoded entirely within the gene for GANP, but intriguingly, the two proteins occupy different locations in the cell and have different activities. Fig 1a shows that MCM3AP is identical to the carboxy-terminal 721 amino acids of GANP. This led Kuwahara *et al.* to propose^4^ reasonably that MCM3AP is a splice variant of GANP. We show here that although the sequence of MCM3AP is identical to the COOH-terminal domain of GANP, the two genes are expressed independently from two different promoters. The promoter for MCM3AP is contained in intron 16 of GANP and is cytokine dependent. Next, we compare the intracellular locations of overexpressed MCM3AP and GANP. We have published evidence that endogenous GANP is concentrated at the nuclear envelope and also present in the nucleus.^7^ However, it is not possible to determine the localisation of endogenous MCM3AP because all its amino acid sequences, and therefore all its epitopes, are also present in GANP, which is expressed at higher levels than MCM3AP. Overexpressed GFP-tagged MCM3AP localises to the cytoplasm and can shuttle between the nucleus and cytoplasm, whereas overexpressed GANP localises to the nucleus and the nuclear envelope, as we reported for endogenous GANP. When MCM3AP and GANP are expressed at lower levels, MCM3AP localises to the nucleus and cytoplasm, whereas GANP localises primarily to the nuclear envelope. We also show that during apoptosis, GANP is cleaved into an N-terminal fragment containing its FG repeat region and Sac3 homology domain and a C-terminal fragment containing its MCM3AP domain and its CID motif. We propose that MCM3AP and GANP should be referred to as independent but overlapping genes and that databases should be revised to reflect this.

### Results and Discussion

GANP contains a domain that is identical to MCM3AP and transcribed from the same locus (Fig. 1a). The nucleotide sequence for MCM3AP is completely contained within the 3′ region of the GANP sequence, which resulted in the suggestion that MCM3AP may be a splice variant of GANP.^4^

To determine whether MCM3AP can be transcribed independently of GANP, we first examined the intron immediately upstream of the first exon of MCM3AP for potential transcription factor binding sites, and observed that NF-IL6, AP-1, NF-κB, and p53 consensus transcription factor binding sites are present immediately upstream of the MCM3AP sequence (Fig. 1b; Supp. Fig. S1c). In contrast, the promoter sequence of GANP had been examined previously and found to contain consensus transcription factor binding sites for E2F, E-box, BSAP, and PU.1,^12^ indicating that GANP and MCM3AP may be regulated differently. Next, we asked whether MCM3AP mRNA could be generated by independent transcription from an MCM3AP-specific promoter by performing 5′ rapid amplification of cDNA ends (5′RACE) using cDNA prepared from HeLa cells. We detected a  200-bp product from two rounds of nested PCR using a primer 70 bases upstream of the first ATG in MCM3AP (Fig. 1c). The PCR product was sequenced and showed a transcriptional initiation start site about 270 bp upstream of the MCM3AP ATG start codon, within exon 17 of GANP and immediately downstream of the cluster of transcription factor consensus binding sites. An identical transcriptional start site, as well as a minor start site, was detected by 5′RACE using cDNA from a different cell line (HEK293) (Supp. Fig. S1a).

To determine whether this region contains intrinsic promoter activity, the putative MCM3AP promoter sequence was cloned into a luciferase vector, transfected into HCT116 colon carcinoma cells, and assayed for activity 48 h after transfection. This region had a level of activity around 3-fold higher than that seen with the empty vector control (Supp. Fig. S1b), suggesting that it contains intrinsic promoter activity.

Furthermore, tumour necrosis factor α (TNFα) and interleukin-6 (IL6), cytokines that can activate signalling pathways, including NF-κB, NF-IL6, and AP-1,^13,14^ enhance the promoter activity. When HEK293 cells were treated with TNFα and IL6, we observed an approximately 7-fold increase in the activity of this region compared to an empty vector control (Fig. 1d). Mutagenesis studies showed that NF-κB, NF-IL6, and AP-1 cooperate to collectively contribute around 60% of the increase in activity of the MCM3AP promoter observed in response to cytokine treatment (Fig. 1e; Supp. Fig. S1d).

Taken together, these results indicate that MCM3AP can be transcribed independently of GANP, although they do not rule out the possibility that MCM3AP mRNA could also arise from a spliced larger transcription product. Additionally, we have shown that MCM3AP and GANP have different functions in the cell,^2,3,7^ although they are both referred to as MCM3AP in the genomic databases. To clarify the confusion between GANP and MCM3AP, we propose that they should be referred to as separate but overlapping genes.

Next, we compared the localisation of MCM3AP with that of GANP. We were unable to determine the localisation of endogenous MCM3AP, as any antibody raised against MCM3AP would also recognise GANP, which is present in greater amounts. Consistent with previous studies,^3^ overexpressed GFP-tagged MCM3AP localised to the cytoplasm and could shuttle between the nucleus and cytoplasm in a leptomycin-B-dependent manner (Fig. 2a). We next determined the localisation of MCM3AP and GANP when expressed at lower levels by generating stable cell lines. In this setting, MCM3AP displayed both nuclear and cytoplasmic staining (Fig. 2b and c). In contrast to MCM3AP, GANP displayed predominantly nuclear envelope staining (Fig. 2b and c). This result agrees with our previous finding demonstrating that endogenous GANP localises to the nuclear envelope with weaker nuclear interior staining.^7^ Thus, MCM3AP and GANP have different locations in the cell.

GANP has been recently shown to be essential for mRNA export in mammalian cells.^7^ As GANP depletion results in cell death, this suggests that GANP may be a prime target for inactivation during apoptosis. We therefore induced apoptosis using a variety of stimuli and examined the levels of GANP by Western blot. We found that a cleavage product of around 110 kDa was generated following treatment with either TNFα or camptothecin (Fig. 3a; Supp. Fig. S2a). Apoptosis was confirmed by the presence of the PARP cleavage product (Fig. 3a). Examination of the GANP amino acid sequence for potential caspase cleavage sites revealed a potential caspase-8 cleavage site that would generate a  110-kDa fragment. To determine whether caspase-8 could mediate GANP cleavage, we performed an *in vitro* cleavage assay using structure-bound fractions from U2OS cells and recombinant caspase-8. Figure 3b shows that recombinant caspase-8 causes cleavage of GANP into a  110-kDa fragment, which has a similar mobility to the GANP fragment generated upon induction of apoptosis. We next mapped the cleavage site of caspase-8 in GANP by N-terminal sequencing of fragments generated following *in vitro* cleavage of recombinant GANP (689–1315) with caspase-8 (Fig. 3c). The fragment generated by cleavage contained the sequence APLSSL, which maps to residues 1024–1029 of full-length GANP (Supp. Fig. S2b), indicating that caspase-8 cleaves GANP once following the VEPD site spanning residues 1020–1023, producing fragments of around 111 kDa and 107 kDa. Therefore, these results indicate that during apoptosis, GANP is cleaved into a fragment that contains the whole MCM3AP sequence. This fragment is ∼30 kDa larger than MCM3AP. More importantly, we do not detect this fragment in normal unstressed cells. Interestingly, the CID motif, which forms a scaffold within TREX-2 to facilitate coupling of transcription and export, is located immediately upstream of the MCM3AP domain and downstream of the caspase-8 cleavage site. We note the presence of an RNA recognition motif in the N-terminal region of GANP, and we are currently investigating the significance of this finding. Therefore, during apoptosis, GANP is cleaved into two separate fragments, one containing the CID motif and the MCM3AP domain and the other containing the N-terminal FG repeat region that interacts with the major mRNA export factor NXF1 and the Sac3 homology domain. Thus, this caspase-8-dependent cleavage of GANP could potentially dismantle the transcription/export machinery during apoptotic cell death by preventing the coupling of transcription (through TREX-2 and SAGA via the CID motif) with mRNA export (through the N-terminal FG repeat region that interacts with NXF1).

Here, we show that although the MCM3AP gene is contained entirely within the larger GANP gene, MCM3AP can be transcribed independently from GANP from a separate promoter. We find that the MCM3AP promoter elements are poorly conserved in mice, suggesting that the regulation of MCM3AP may be human specific; however, this is not surprising, as Odom *et al.* have shown that most transcription factor binding events within 5 kb of a transcription start site are species specific when comparing mouse and human genes.^15^ More importantly, the MCM3AP initiating methionine is conserved throughout vertebrates. Although MCM3AP is an inhibitor of DNA replication initiation whereas GANP is required for mRNA export in mammalian cells, they are both recognised as MCM3AP in the genomic databases. Therefore, to avoid further confusion, they should now be referred to as separate but overlapping genes. This report is an interesting example in nature of two separate gene products from the same locus that perform two entirely different functions in the cell.

The following are the supplementary materials related to this article.

Supplementary Figure 1 MCM3AP transcription start site mapping. (A) 5’RACE in HEK293 cells. 5’RACE was performed in HEK293 cells according to the manufacturers instructions (Ambion). A control reaction without Tobacco Acid Pyrophosphatase (TAP) was carried out to measure the efficiency of calf intestinal phosphatase treatment and to ensure that mature mRNA was used in the 5’RACE reaction. A 2 round nested PCR reaction was performed to identify the MCM3AP transcription initiation start site. The 1st round was performed with primers homologous to the 5’ RACE adapter sequence and to a region 50 bp downstream of the MCM3AP start codon. The 2nd round was performed using template from the 1st round with a different primer homologous to a region 70 bp upstream of the start codon. Products from the 2nd round of a nested PCR reaction are shown. (B) MCM3AP promoter sequence contains intrinsic activity. HCT116 cells were co-transfected with pGL3 luciferase plasmid with MCM3AP promoter sequence (or empty vector) and a constant amount of renilla luciferase control plasmid, pRL-TK using Polyfect (Qiagen). Cells were harvested 48 hours post-transfection and assayed for luciferase activity (Promega). The firefly/renilla luciferase ratio was then calculated. (C) Promoter sequence of MCM3AP is indicated. Putative transcription factor binding sites are underlined and were detected using Genetyx version 10 software. (D) NF-IL6 and NF-kB contribute to cytokine-mediated increase in promoter activity. Mutations of the NF-IL6 site (TTTTGAAAT to TTTTGACCC) and each NF-kB site (GGGGTTTCAC to CCCGTTTCAC) were made using the Quik-Change mutagenesis kit (Stratagene). The mutated promoter sequences were cloned into the pGL3 vector and sequenced for confirmation of the mutations. These plasmids were then transfected into HEK293 cells following cytokine treatment and assayed for luciferase activity as above. Note that single transcription factor binding site mutants caused no significant reduction in cytokine-mediated increase in promoter activity.

Supplementary Figure 2 GANP is cleaved during apoptosis by caspase-8 at VEPD site spanning residues 1020–1023. (A) GANP is cleaved following camptothecin treatment. HCT116 cells were treated with camptothecin (10 μM) (Sigma) for the indicated times to induce apoptosis. Structure-bound samples were analysed by western blot with the indicated antibodies. (B) Representation of Caspase-8 cleavage site in GANP. N-terminal sequencing of fragment generated *in in-vitro* caspase-8 cleavage assay revealed that caspase-8 cleaves GANP at VEPD site spanning residues 1020-1023.

## Figures and Tables

**Fig. 1:**
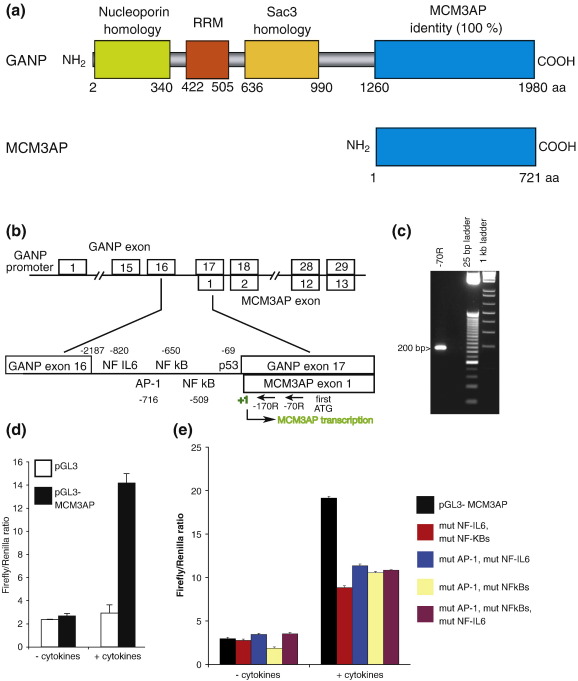
MCM3AP can be transcribed independently of GANP. (a) Domain organization of MCM3AP and GANP. GANP protein contains a domain that is identical to MCM3AP and transcribed from the same sequence. GANP also contains a region of homology to Sac3, a yeast mRNA export factor, and to nucleoporins, as well as a putative RNA recognition motif (RRM). (b) The promoter region of MCM3AP contains putative transcription factor binding sites for NF-IL6, AP-1, NF-κB, and p53. Diagram of the human GANP and MCM3AP genes. Exons 2–13 of MCM3AP are the same as exons 18–29 of GANP. MCM3AP exon 1 starts in GANP exon 17. A cluster of transcription factor binding sites was detected in GANP intron 16 using Genetyx version 10 software.  (c) 5′RACE identifies a transcription initiation site of MCM3AP within exon 17 of GANP. Two rounds of nested PCR were performed to identify the transcriptional start site of MCM3AP using pre-prepared RACE-ready cDNA from HeLa cells (Invitrogen). The first round was performed with primers homologous to the 5′RACE adapter sequence and to a region 50 bp downstream of the MCM3AP start codon. The second round was performed using the template from the first round with a different primer homologous to a region 70 bp upstream of the start codon. Products from the second round of a nested PCR reaction are shown. Transcription initiation site is indicated in green in (b). (d) MCM3AP promoter is cytokine responsive. Subconfluent HEK293 cells were treated or mock-treated with TNFα (Calbiochem) and IL6 (Calbiochem) and transfected with pGL3 (Promega) or pGL3-MCM3AP promoter and a constant amount of renilla luciferase control plasmid, pRL-TK (Promega) using Polyfect (Qiagen). Cells were harvested 40 h after transfection and assayed for luciferase activity (Promega). The firefly/renilla luciferase ratio was then calculated. Average results from three experiments are shown. (e) AP-1, NF-IL6, and NF-κB contribute to cytokine-mediated increase in promoter activity. Mutations of the AP-1 site (TGAGTAG to TGAGCCT), NF-IL6 site (TTTTGAAAT to TTTTGACCC), and each NF-κB site (GGGGTTTCAC to CCCGTTTCAC) were made using the QuikChange mutagenesis kit (Stratagene). The mutated promoter sequences were cloned into the pGL3 vector and sequenced for confirmation of the mutations. These plasmids were then transfected into HEK293 cells following cytokine treatment and assayed for luciferase activity as above.

**Fig. 2:**
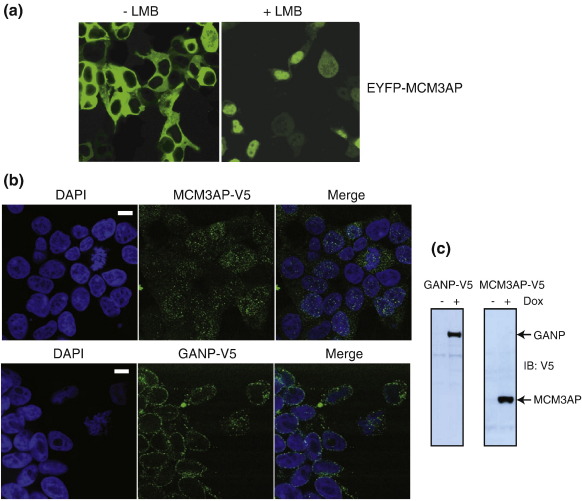
MCM3AP and GANP have different localisations in the cell. (a) MCM3AP can shuttle between the cytoplasm and nucleus. 293 T cells were transfected with EYFP-MCM3AP plasmid using Lipofectamine 2000 (Invitrogen) according to the manufacturer's protocol. Twenty-four hours after transfection, cells were treated with leptomycin B for 90 min and analysed by immunofluorescence as described previously.^7^ (b) MCM3AP localises to the nucleus and cytoplasm, whereas GANP localises to the nuclear envelope. MCM3AP and GANP stable cell lines were generated as follows. Full-length GANP or MCM3AP sequence was amplified by PCR, adding KpnI and XhoI restriction enzyme ends. The KpnI site was designed to create a Kozak sequence around the start codon in both cases. The PCR fragment was ligated into pUB6/V5-His A (Invitrogen) in the correct reading frame to add C-terminal V5 and poly-His tags. Then, KpnI and PmeI were used to excise the protein and tags to ligate into pcDNA5/FRT/To (Invitrogen), which had been mutated to remove an upstream PmeI site. This construct was co-transfected with pOG44 into Flp-In TREx 293 cells according to the manufacturer's protocol (Invitrogen). This after antibiotic selection produces a cell line that can be induced to express GANP or MCM3AP by the addition of 0.1 mg/ml doxycycline. GANP and MCM3AP localisations were determined by immunofluorescence with anti-V5 antibody (Invitrogen). (c) Expression of GANP-V5 and MCM3AP-V5 were validated by Western blot using anti-V5 antibody.

**Fig. 3:**
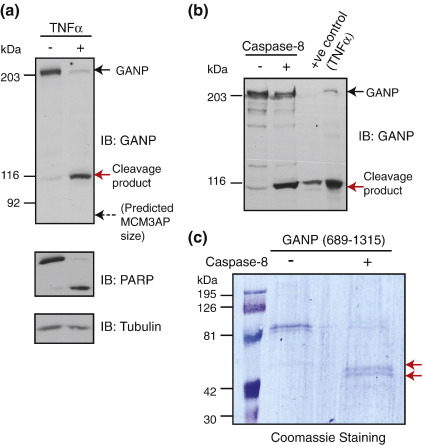
GANP is cleaved during apoptosis into a fragment that still contains the MCM3AP sequence. (a) HEK293 cells were treated with TNFa and analysed by Western blotting with antibodies against GANP, PARP (Calbiochem), and tubulin (Abcam). The cleavage product is ∼30 kDa larger than MCM3AP and indicated by a red arrow. Dashed arrow indicates the predicted position of MCM3AP. (b) Recombinant caspase-8 cleaves GANP into a  110-kDa fragment. Structure-bound fraction from HEK293 cells was incubated with recombinant caspase-8 (Calbiochem) for 1 h at 30 °C and analysed by Western blotting with an anti-GANP antibody. (c) Mapping of caspase-8 cleavage site. A fragment of GANP spanning residues 689–1315 was expressed and purified in *E. coli*, and 100 μg was incubated with recombinant caspase-8 in an *in vitro* cleavage reaction under identical experimental conditions to the *in vivo* reaction above. Samples were analysed by SDS-PAGE and Coomassie staining, and N-terminal sequencing was carried out on the bands by the University of Cambridge Department of Biochemistry to determine the identity of the cleavage site.

## References

[1] Takei Y., Tsujimoto G. (1998). Identification of a novel MCM3-associated protein that facilitates MCM3 nuclear localisation. J. Biol. Chem..

[2] Takei Y., Swietlik M., Tanoue A., Tsujimoto G., Kouzarides T., Laskey R. (2001). MCM3AP, a novel acetyltransferase that acetylates replication protein MCM3. EMBO Rep..

[3] Takei Y., Assenberg M., Tsujimoto G., Laskey R. (2002). The MCM3 acetylase MCM3AP inhibits initiation, but not elongation, of DNA replication via interaction with MCM3. J. Biol. Chem..

[4] Abe E., Kuwahara K., Yoshida M., Suzuki M., Terasaki H., Matsuo Y. (2000). Structure, expression and chromosomal localisation of the human gene encoding a germinal centre-associated nuclear protein (GANP) that associates with MCM3 involved in the initiation of DNA replication. Gene.

[5] Fujimura S., Xing Y., Takeya M., Yamashita Y., Ohshima K., Kuwahara K., Sakaguchi N. (2005). Increased expression of germinal center-associated nuclear protein RNA-primase is associated with lymphomagenesis. Cancer Res..

[6] Kuwahara K., Fujimura S., Takahashi Y., Nakagata N., Takemori T., Aizawa S., Sakaguchi N. (2004). Germinal center-associated nuclear protein contributes to affinity maturation of B cell antigen receptor in T cell-dependent responses. Proc. Natl Acad. Sci..

[7] Wickramasinghe V.O., McMurtrie P.I., Mills A.D., Takei Y., Penrhyn-Lowe S., Amagase Y. (2010). mRNA export from mammalian cell nuclei is dependent on GANP. Curr. Biol..

[8] Fischer T., Strasser K., Racz A., Rodriguez-Navarro S., Oppizzi M., Ihrig P. (2002). The mRNA export machinery requires the novel Sac3p–Thp1p complex to dock at the nucleoplasmic entrance of the nuclear pores. EMBO J..

[9] Ciccarelli F.D., Izaurralde E., Bork P. (2003). The PAM domain, a multi-protein complex-associated module with an all-alpha-helix fold. BMC Bioinformatics.

[10] Faza M.B., Kemmler S., Jimeno S., Gonzalez-Aguilera C., Aguilera A., Hurt E., Panse V.G. (2009). Sem1 is a functional component of the nuclear pore complex-associated messenger RNA export machinery. J. Cell Biol..

[11] Jani D., Lutz S., Marshall N.J., Fischer T., Kohler A., Ellisdon A.M. (2009). Sus1, Cdc31, and the Sac3 CID region form a conserved interaction platform that promotes nuclear pore association and mRNA export. Mol. Cell.

[12] El-Gazzar M., Maeda K., Nomiyama H., Nakao M., Kuwahara K., Sakaguchi N. (2001). PU.1 is involved in the regulation of B lineage-associated and developmental stage-dependent expression of the germinal center-associated DNA primase GANP. J. Biol. Chem..

[13] Akira S. (1997). IL-6-regulated transcription factors. Int. J. Biochem. Cell Biol..

[14] Baud V., Karin M. (2001). Signal transduction by tumor necrosis factor and its relatives. Trends Cell Biol..

[15] Odom D.T., Dowell R.D., Jacobsen E.S., Gordon W., Danford T.W., MacIsaac K.D. (2007). Tissue-specific transcriptional regulation has diverged significantly between human and mouse. Nat. Genet..

