# Supplementary figures and images for: MCM3AP Is Transcribed from a Promoter within an Intron of the Overlapping Gene for GANP
Source: J Mol Biol. 2011 Feb 25;406(3):355–61. doi: 10.1016/j.jmb.2010.12.035 (PMC3121959; doi:10.1016/j.jmb.2010.12.035)

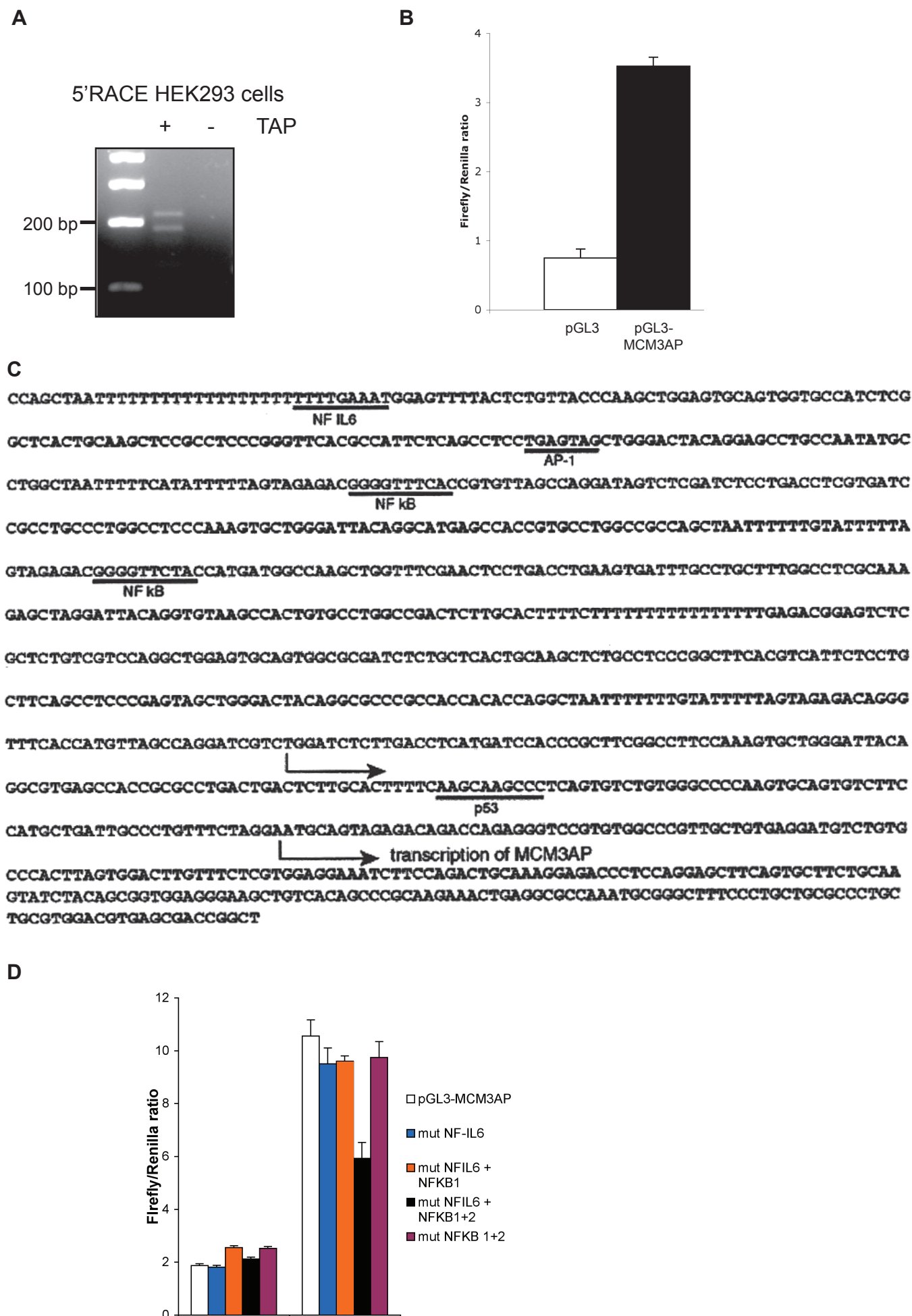

Supp Figure 1- MCM3AP transcription start site mapping

Supplement: Supplementary Figure 1 — MCM3AP transcription start site mapping. (A) 5’RACE in HEK293 cells. 5’RACE was performed in HEK293 cells according to the manufacturers instructions (Ambion). A control reaction without Tobacco Acid Pyrophosphatase (TAP) was carried out to measure the efficiency of calf intestinal phosphatase treatment and to ensure that mature mRNA was used in the 5’RACE reaction. A 2 round nested PCR reaction was performed to identify the MCM3AP transcription initiation start site. The 1st round was performed with primers homologous to the 5’ RACE adapter sequence and to a region 50 bp downstream of the MCM3AP start codon. The 2nd round was performed using template from the 1st round with a different primer homologous to a region 70 bp upstream of the start codon. Products from the 2nd round of a nested PCR reaction are shown. (B) MCM3AP promoter sequence contains intrinsic activity. HCT116 cells were co-transfected with pGL3 luciferase plasmid with MCM3AP promoter sequence (or empty vector) and a constant amount of renilla luciferase control plasmid, pRL-TK using Polyfect (Qiagen). Cells were harvested 48 hours post-transfection and assayed for luciferase activity (Promega). The firefly/renilla luciferase ratio was then calculated. (C) Promoter sequence of MCM3AP is indicated. Putative transcription factor binding sites are underlined and were detected using Genetyx version 10 software. (D) NF-IL6 and NF-kB contribute to cytokine-mediated increase in promoter activity. Mutations of the NF-IL6 site (TTTTGAAAT to TTTTGACCC) and each NF-kB site (GGGGTTTCAC to CCCGTTTCAC) were made using the Quik-Change mutagenesis kit (Stratagene). The mutated promoter sequences were cloned into the pGL3 vector and sequenced for confirmation of the mutations. These plasmids were then transfected into HEK293 cells following cytokine treatment and assayed for luciferase activity as above. Note that single transcription factor binding site mutants caused no significant reduction in cytokine-mediated i [file mmc1.pdf]
