# Supplementary material for: MCM3AP Is Transcribed from a Promoter within an Intron of the Overlapping Gene for GANP
Source: J Mol Biol. 2011 Feb 25;406(3):355–61. doi: 10.1016/j.jmb.2010.12.035 (PMC3121959; doi:10.1016/j.jmb.2010.12.035)
Supplement: Supplementary Figure 2 — GANP is cleaved during apoptosis by caspase-8 at VEPD site spanning residues 1020–1023. (A) GANP is cleaved following camptothecin treatment. HCT116 cells were treated with camptothecin (10 μM) (Sigma) for the indicated times to induce apoptosis. Structure-bound samples were analysed by western blot with the indicated antibodies. (B) Representation of Caspase-8 cleavage site in GANP. N-terminal sequencing of fragment generated in in-vitro caspase-8 cleavage assay revealed that caspase-8 cleaves GANP at VEPD site spanning residues 1020-1023. [file mmc2.pdf]

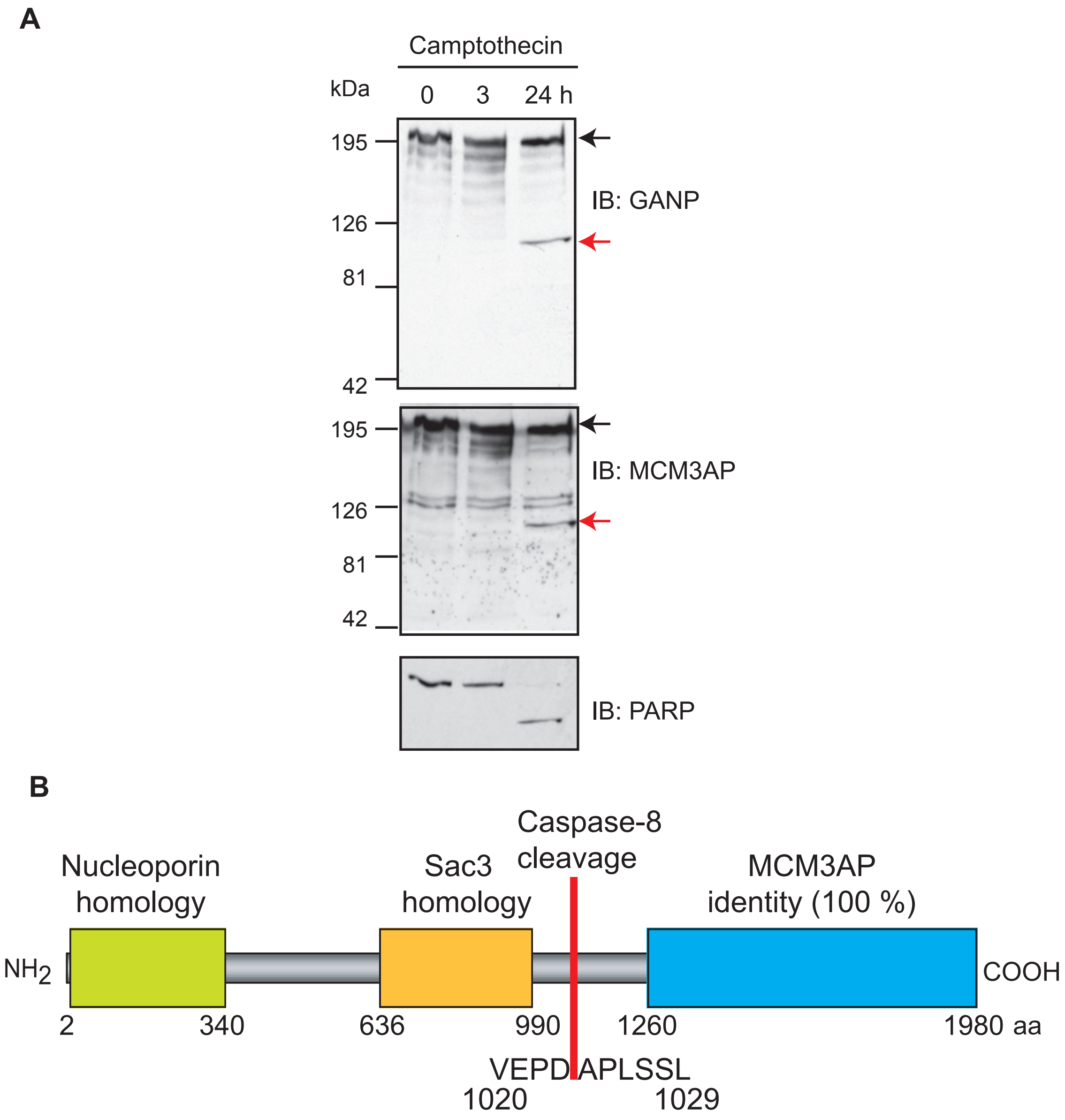

Supplementary Figure 2- GANP is cleaved during apoptosis by Caspase-8 at VEPD site spanning residues 1020-1023
